# Universal coronavirus vaccines: the time to start is now

**DOI:** 10.1038/s41541-020-0198-1

**Published:** 2020-05-28

**Authors:** Luca T. Giurgea, Alison Han, Matthew J. Memoli

**Affiliations:** LID Clinical Studies Unit, Laboratory of Infectious Diseases, Division of Intramural Research, National Institute of Allergy and Infectious Diseases, National Institutes of Health, Bethesda, MD 20892 USA

**Keywords:** Vaccines, Viral infection, SARS-CoV-2, Influenza virus

## Abstract

The continued explosive spread of severe acute respiratory syndrome coronavirus 2 (SARS-CoV-2) despite aggressive public health measures has triggered an unprecedented international vaccine effort. However, correlates of protection, which can help guide intelligent vaccine design, are not known for SARS-CoV-2. Research on influenza immunity and vaccine development may provide valuable lessons for coronavirus efforts, especially considering similarities in rapid evolutionary potential. The apparent inevitability of future novel coronavirus outbreaks must prompt work on a universal coronavirus vaccine.

The severe acute respiratory syndrome coronavirus 2 (SARS-CoV-2) pandemic continues to spread throughout the world with explosive outbreaks underway throughout much of Europe and the United States. Even though the pandemic has slowed down in some countries, other nations are still experiencing a rapid rise in cases and their healthcare systems are pushed to the limits^1^. The case fatality ratio (CFR) varies widely by country and while population characteristics and access to healthcare are critically important factors, differences in the extent of testing of mild cases are likely responsible for some of the observed variation in the CFR through effects on the denominator. Interestingly, in the hardest hit areas, only a small fraction of the population has been reported to be infected. It is unclear how much of this is due to asymptomatic clinical presentations, testing availability, viral transmission dynamics, changes in individual behavior, and aggressive isolation and quarantine actions. Some experts suspect a significant number of mild or asymptomatic infections go undiagnosed, leading to a falsely elevated CFR and making the pandemic much more difficult to control in the setting of asymptomatic transmission^2^. Public health measures helped to extinguish the SARS outbreak 17 years ago, but so far, they have only been able to slow down the coronavirus disease 2019 (Covid-19) pandemic^3^.

In order to combat the disease, governments, international organizations, private companies, and academic institutions across the planet have launched a multifaceted vaccine development response with unprecedented rapidity. At least 110 vaccine candidates are in preclinical testing and at least 5 vaccines have already begun early clinical testing^4^. The focus of the response is haste, understandably so, leading to use of novel technologies, including DNA plasmids, viral vectors, virus-like particles, Baculovirus/mammalian expression systems, and mRNA vaccines among others. Many of these utilize sequencing information that was made available quickly after the onset of the outbreak and can be developed more rapidly than classical vaccine preparation strategies utilizing live virus as substrate, which necessitate the use of cumbersome biosafety protection measures^5^. The purpose of most early candidate vaccines is development of antibodies against the Spike glycoprotein (S protein), which have been implicated in protection against SARS-CoV in animal models and have been found in SARS-COV and SARS-CoV-2 convalescent patient sera^6–8^. However, we do not yet have evidence to show that anti-S protein antibodies are protective in humans against SARS-CoV-2, and if they are, how much protection is afforded. Recent data have shown 94% of patients who recover from mild Covid-19 have neutralizing antibodies 2 weeks after symptom onset, but neutralizing antibody titers correlate only moderately with measured anti-S protein antibody^9^. Careful study of potential patients with SARS-CoV-2 reinfection may help shed light on this question, but cases have not been documented in the literature. Reports may be confounded by prolonged RNA shedding, which can persist over 30 days in almost 10% of patients, regardless of resolution of symptoms^10^. The viability of virus associated with prolonged shedding and consequently the infectivity of these patients is also unknown, considering that in a separate study of nine patients, live SARS-CoV-2 was isolated only up to day 7 despite ongoing high viral loads by reverse transcription polymerase chain reaction^11^. The distinction between viable virus and RNA shedding may have ramifications on study of immune correlates of viral clearance.

Vaccine development against respiratory viruses is a challenging task. Currently, the only approved vaccines against respiratory viruses are those against influenza. Despite decades of research, modern vaccines have had limited effectiveness against seasonal influenza and are unlikely to provide broad protection against novel influenza strains capable of causing pandemics^12,13^. Only in the past few years, a century after the advent of the 1918 influenza pandemic that was one of the most catastrophic events in human history responsible for 50–100 million deaths, a concerted effort was initiated to accelerate the development of a universal influenza vaccine^13,14^. Many unknowns exist with respect to SARS-CoV-2, but research into influenza immunity and vaccine development may provide valuable lessons for Covid-19 and may help guide vaccination strategies. We have known for decades that antibodies against hemagglutinin (the dominant surface protein of the influenza particle) at a titer of 1:40 or greater are associated with >50% reduction in disease, but more recent data have suggested antibody against influenza neuraminidase, another viral surface protein, may be a superior predictor of clinical outcomes^15,16^. We have no such knowledge of correlates of protection against coronaviruses, and specifically against SARS-CoV-2, thereby making the process of developing an effective vaccine that much more difficult. Interestingly, the importance of anti-neuraminidase immunity in protection against influenza was recognized during the H3N2 pandemic. Through serendipitous ongoing trials, researchers were able to seize an opportunity to obtain preinfection and postinfection sera demonstrating the ability of anti-neuraminidase antibodies to reduce infection rates^17^. We need to obtain similar information for SARS-CoV-2 to help guide and refine vaccine development.

Both influenza and coronaviruses have an inherent capacity for rapid mutation due to the low fidelity of RNA polymerase and a capability to form novel viruses through reassortment with a vast reservoir of zoonotic viruses^13,18,19^. Influenza surface proteins have non-conserved regions which can accommodate mutations that limit the effect of antibodies without impairing enzyme activity (antigenic drift), potentially leading to loss of immunity in infected or vaccinated individuals from one season to the next^20^. Genetic drift of spike protein has been demonstrated in endemic human coronavirus OC43, suggesting mutations can be accumulated in non-conserved regions over time^21^. Furthermore, an escape mutation conferring resistance to neutralizing antibody was found in vitro after applying immune pressure to 2002/2003 SARS-CoV. Interestingly, this mutation was also found to have occurred naturally in 2003/2004 SARS-CoV strains raising the possibility that SARS-CoV-2 has the potential to evolve resistance against antibodies induced by some vaccine strategies currently under development^18^. Genetic differences within the receptor-binding domain (RBD) discriminating two major types of circulating SARS-CoV-2 indicate that the virus is already adapting^22^. Targeting of conserved epitopes has been proposed as a strategy to combat influenza’s adaptive capabilities and may be a potential strategy against SARS-CoV-2 as well. Amino acid sequence similarity between SARS-CoV S protein and SARS-CoV-2 S protein has been estimated only at 77% with significant changes present in the RBD^19,23^. In this context, many antibodies against SARS-CoV S protein have not shown promising SARS-CoV-2 binding and neutralizing capacity^24–26^. Similarly, antibodies against SARS-CoV-2 RBD have not shown cross-reactivity to SARS-CoV RBD or Middle East respiratory syndrome-CoV RBD^24^. Recent work has clarified that, despite only 46.7% protein sequence similarity between the SARS-CoV S1_B_ receptor-binding subdomain and the SARS-CoV-2 S1_B_ receptor-binding subdomain, the S1_B_ RBD core domain is highly conserved^27^. Antibodies have been identified targeting epitopes in S1_B_ RBD core domain, which show cross-reactive potential against both SARS-CoV and SARS-CoV-2^23,24,27^. These findings may help guide development of vaccines with broader coverage, though the protective effects of such antibodies have yet to be demonstrated in animal models or human studies.

An additional problem is the waning of antibody titers over time after vaccination or recovery from live infection. Consequently, individuals are left vulnerable to reinfection, as demonstrated in influenza, even with an identical strain during the next season^28^. Similarly, antibodies against S protein diminished over time in SARS survivors^29^. We are then faced with a critically important but unanswered question: For how long will SARS-CoV-2 circulate? Whereas the SARS outbreak was extinguished, all five characterized pandemic influenza viruses established endemicity, facilitated, at least in part, by antigenic drift and waning antibody titers. Though impossible to predict with certainty, the prospect of SARS-CoV-2 also establishing endemicity is one the medical community needs to be prepared for, including the potential for accumulation of mutations in the face of increasing immune pressure. Considering that three coronavirus outbreaks have occurred in the past two decades, even if SARS-CoV-2 disappears in a similar fashion to SARS-CoV, it seems inevitable that another novel coronavirus outbreak will occur in the future. Acknowledging the limitations in cross-protection of most SARS-CoV-induced antibodies against SARS-CoV-2, it is quite likely that vaccines tailored to SARS-CoV-2 would not offer protection against novel coronaviruses^25,26^. Even with advances in the rapidity of vaccine development technology, the delay between epidemic onset and implementation of a novel vaccine candidate in the community is responsible for immense societal costs.

After the SARS outbreak, we lost interest and failed to complete development of a vaccine for use in case of a recurrent outbreak. We must not make the same mistake again. Instead we must go further. We must work toward a universal coronavirus vaccine with broad protection against a diverse number of coronaviruses. We need to understand the correlates of protection against coronaviruses and improve our understanding of their pathogenesis, transmission, and viral evolution. These studies will be absolutely necessary for us to intelligently design efficacious vaccines not just against SARS-CoV-2 but also against other coronaviruses that are likely to emerge in the future. If work on influenza can serve as a lesson, a universal coronavirus vaccine will not be easy to develop, but if we do not start work on one now, we will find ourselves in a similar situation during the next outbreak.
